# Effects of Degree of Urbanization and Lifetime Longest-Held Occupation on Cognitive Impairment Prevalence in an Older Spanish Population

**DOI:** 10.3389/fpsyg.2017.00162

**Published:** 2017-02-13

**Authors:** Laura Lorenzo-López, José C. Millán-Calenti, Rocío López-López, Clara Diego-Diez, Blanca Laffon, Eduardo Pásaro, Vanessa Valdiglesias, Ana Maseda

**Affiliations:** ^1^Universidade da Coruña, Gerontology Research Group, Instituto de Investigación Biomédica de A Coruña (INIBIC), Complexo Hospitalario Universitario de A Coruña (CHUAC), SERGAS, A CoruñaSpain; ^2^Universidade da Coruña, DICOMOSA Group, Department of Psychology, Area of Psychobiology, Faculty of Education Sciences, Campus Elviña s/n, A CoruñaSpain

**Keywords:** urbanization, rurality, occupation, cognition, elderly

## Abstract

Our aim was to estimate the prevalence of cognitive impairment in rural and urban elderly populations and to examine the relationship between lifetime occupation and general cognitive performance. A cross-sectional study was carried out covering a representative sample (*n* = 749) of adults aged ≥65 years. Two categories were created to define the degree of urbanization using a criterion of geographical contiguity in combination with a minimum population threshold: densely populated (urban) areas and intermediate-thinly populated (rural) areas. Occupational histories were ranked by skill level requirements according to the Spanish National Classification of Occupations. Prevalence estimates of cognitive impairment were measured with the Mini-Mental State Examination. Results show that rural residence was not significantly associated with higher risk of cognitive impairment. A protective effect of cognitive demands at work against age-related cognitive decline was observed. However, this effect was not independent of confounder factors, such as age and education. A low overall prevalence of cognitive impairment was observed (6.5%), compared with previous estimates, possibly due to the sample selection in senior centers. Occupation during active life is not an isolated protective factor against cognitive impairment, and it is closely related to educational level. In future geriatric programs, description of both factors should be taken into consideration in screening older adults at increased risk of cognitive impairment and dementia.

## Introduction

The study of the prevalence of cognitive impairment is essential since it is a common condition in elderly and has a complex relationship with more serious conditions, such as dementia or depression (Steffens et al., 2014).

Previous research on the prevalence of age-related cognitive impairment shows considerable variability, due to the different diagnostic criteria used, the degree of severity of clinical manifestations, and the age range covered (Brayne et al., 1998; Rait et al., 2005). The recognition of the characteristics of the current cohort of older adults in Spain, including the study of the prevalence of cognitive impairment, is crucial for an adequate planning of public health strategies to this population, because Spain has one of the highest rates of aging (high level of life expectancy at birth) in the world (United Nations, Department of Economic, and Social Affairs, Population Division, 2015), and cognitively impaired elders are likely to experience different degrees of physical impairment and/or chronic diseases, and serious functional limitations on basic and/or instrumental daily activities (Millán-Calenti et al., 2012).

Besides the well-recognized age and education effects, patterns of cognitive impairment have been shown to be determined by geographical/rurality variables. In fact, the prevalence of cognitive impairment has been shown to be higher in rural than in urban populations (Martín Lesende et al., 2001; Nunes et al., 2010). The better functional-cognitive status observed in the urban environment has been, in general, associated with differences in lifestyle and greater accessibility and availability of the social-health care resources (Martín Lesende et al., 2001). Finding possible differences in the risk of cognitive impairment among geographic areas may be important in the allocation of public resources in rural and urban areas (Keefover et al., 1996); however, the link between the degree of urbanization (DEGURBA) and cognitive impairment has not been widely studied.

Previous studies have also shown that the complexity of primary lifetime occupation may be reflected in cognitive functioning in advancing age (Andel et al., 2005; Correa Ribeiro et al., 2013), revealing a significant association between high mental demands at work and better cognitive functioning in old age (Bosma et al., 2003; Andel et al., 2005; Fisher et al., 2014; Then et al., 2014). High levels of formal education and engagement in stimulating cognitive activities have also been associated with lower levels of age-related cognitive impairment (Jorm et al., 1994; Baldivia et al., 2008; Sando et al., 2008). These factors, which seemingly have protective effects, have been termed “cognitive reserve” (Scarmeas and Stern, 2003; Stern, 2012). A recent cognitive reserve structural model has been proposed grouping the main cognitive reserve indicators into two latent variables: educational level (including occupation attainment) and lifestyle (Lojo-Seoane et al., 2014). The role of occupation as cognitive reserve proxy in elderly has also been confirmed in neuroimaging studies (Staff et al., 2004). Importantly, it has been suggested that reserve is dynamic or changeable through the life course, especially in early life (Fritsch et al., 2007).

To our knowledge, the effects of occupation on general cognitive performance in later life have been no previously investigated in a Spanish representative population, and none of the previous studies examining the prevalence of cognitive impairment differentiated between rural and urban areas. With this background, a cross-sectional study was conducted covering a representative sample of older adults. The objectives were: (a) to determine the overall prevalence of cognitive impairment in a large geographically representative elderly population, (b) to compare the prevalence rates in rural and urban settings, and (c) to investigate the influence of principal lifetime occupation in the general cognitive performance at old age. Investigating these points and identifying proxies possibly influencing cognitive reserve in later life can give important insights into protective and risk factors associated with cognitive impairment and may contribute to more effective preventive interventions among older adults.

## Materials and Methods

### Participants

Data were used from baseline assessments from the VERISAÚDE (Effectiveness of the Comprehensive Gerontological Assessment and longitudinal follow-up in the healthy aging promotion) project, which is a large longitudinal study covering a sample of 749 community-dwelling subjects representative of Galician population aged 65 years and older attending senior centers. The distribution of the sample by age and sex was similar to that of the entire Galician elderly population, according to the municipal register of the 2011 National Health Survey (National Statistics Institute, 2011). The level of confidence was 95%, accuracy ±4%, and estimation for data losses 20%. Pearson’s correlation (*r*) determined very strong significant correlations between the distributions of total population vs. total sample (*r* = 0.984, *P* < 0.001); male population vs. male sample (*r* = 0.993, *P* < 0.001); and female population vs. female sample (*r* = 0.973, *P* < 0.001) (see **Table 1**).

**Table 1:** Distributions of the population and sample by age groups and gender, Galicia 2011.

	Population, *n* (%)			Sample-749, *n* (%)		
		
Age group (years)	Total (*n* = 632381)	Males (*n* = 265724)	Females (*n* = 366657)	Total (*n* = 749)	Males (*n* = 295)	Females (*n* = 454)
65–69	160341 (25.36)	75914 (28.57)	84427 (23.03)	181 (24.17)	81 (27.46)	100 (22.03)
70–74	126758 (20.04)	57309 (21.57)	69449 (18.94)	167 (22.30)	67 (22.71)	100 (22.03)
75–79	144228 (22.81)	61378 (23.10)	82850 (22.60)	169 (22.56)	65 (22.03)	104 (22.91)
80–84	105473 (16.68)	41105 (15.47)	64368 (17.56)	124 (16.56)	46 (15.59)	78 (17.18)
85–89	62348 (9.86)	21011 (7.91)	41337 (11.27)	82 (10.95)	28 (9.49)	54 (11.89)
≥90	33233 (5.26)	9007 (3.39)	24226 (6.61)	26 (3.47)	8 (2.71)	18 (3.96)


*Source: Population data (Galician Statistics Institute, 2011).*

From October 2013 through March 2014, a Comprehensive Gerontological Assessment was conducted. The global cognitive status was assessed by qualified clinical psychologists using the Spanish version of the Mini-Mental State Examination (MMSE; Folstein et al., 1975; Blesa et al., 2001). MMSE scores, ranging from 0 to 30, were adjusted for age and level of education, and participants were considered as cognitively impaired if they scored ≤24 (Blesa et al., 2001). The reasons for demographic (age and education) adjustments for the MMSE scores were that it has been demonstrated that both factors influence MMSE scores (Blesa et al., 2001), and that adjusted scores improve the diagnostic classification accuracy of cognitive impairment over unadjusted scores in Spanish older adults (Millán-Calenti et al., 2009). We specifically used the correction method of Blesa et al. (2001), who performed a test correction through a study of linear regression in which the score to be corrected was considered as a dependent variable and age and education as independent variables, according to a specific formula. The corrections add or subtract up to two points depending on age and educational level (see the formula and the correction table of MMSE by age and education in Blesa et al., 2001).

Information on age, sex, and education was self-reported. Educational level was categorized into three levels according to years of formal education: ≤8 years, 9–17 years, and >17 years. The study protocol was approved by the Ethics Committee of the University of A Coruña and was in conformity with the principles embodied in the Declaration of Helsinki. Before the data collection, all participants were informed about the study and signed the corresponding informed consent form.

The manuscript was written according to the STrengthening the Reporting of OBservational studies in Epidemiology (STROBE) statement (Vandenbroucke et al., 2014; von Elm et al., 2014).

### Definition of Urban and Rural: Degree of Urbanization

**FIGURE 1:**
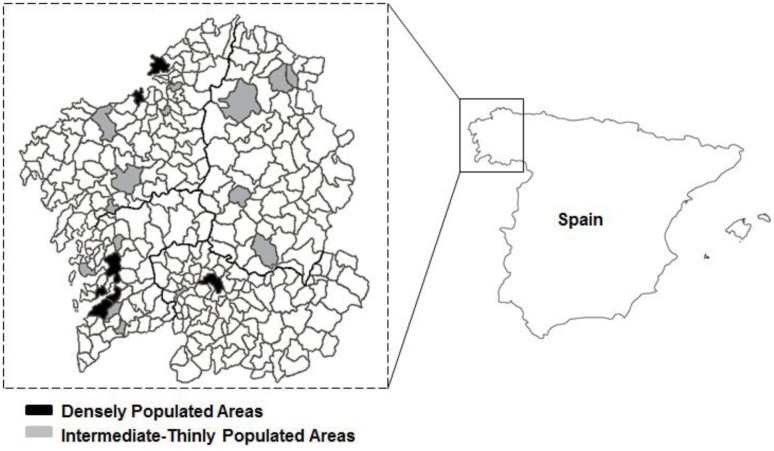
**Map of Galicia (Northwest Spain) showing the urban/rural areas (shaded) included in the study**.

The concept of DEGURBA was defined by the Task Force on Core Social Variables group (Eurostat Task Force on Core Social Variables, 2007), and provides a classification indicating the character of a specific area. For international comparability, two unique area types were defined, using a criterion of geographical contiguity in combination with a minimum population threshold, based on the share of local population living in urban clusters and in urban centers, and 2001 census data (Galician Statistics Institute, 2011): densely populated areas (DPA; cities/large urban areas; seven municipalities), and intermediate-thinly populated areas (ITPA; towns and suburbs and rural areas, 15 municipalities). **Figure 1** presents a map of Galicia showing the urban/rural areas (shaded) included in the study.

### Coding and Classification of Principal Lifetime Occupations

Self-reported information on the participants’ principal lifetime occupation was obtained asking the question: “What occupation/job did you have during the major part of your working life?” Only the longest-held occupation was considered. In order to explore the link between occupation category and cognitive impairment, we first matched the participants’ occupations to the Spanish National Classification of Occupations (CNO-11) (National Statistics Institute, 2012), which is adapted to the International Standard Classification of Occupations (ISCO-08) (International Labour Organization [ILO], 2012), ensuring comparability and coherence between data on occupations from the EU Member States and the rest of the world. CNO-11 hierarchically classified occupations into 10 major groups (1: Managers, 2: Professionals, 3: Technicians and associate professionals, 4: Clerical support workers, 5: Services and sales workers, 6: Skilled agricultural, forestry and fishery workers, 7: Craft and related trades workers, 8: Plant and machine operators, and assemblers, 9: Elementary occupations, 0: Armed forces occupations). In a second step, we classified these 10 major occupation groups in four categories depending on the skill level or the complexity and range of the tasks and duties involved (as described in ISCO-08) (International Labour Organization [ILO], 2012). Specifically, skill levels were coded from 1 (basic skills) to 4 (advanced/specialized skills). Occupations requiring the performance of simple and routine physical or manual tasks (major group 9) were coded as skill level 1 (low, basic). Occupations typically involving the performance of tasks, such as operating machinery and electronic equipment; driving vehicles; maintenance and repair of electrical and mechanical equipment; and manipulation, ordering and storage of information (major groups 4–8) were coded as skill level 2 (middle-low). Occupations involving the performance of complex technical and practical tasks that require an extensive body of knowledge in a specialized field (major group 3) were coded as skill level 3 (middle-high). Occupations typically involving the performance of tasks that require complex problem solving, reasoning, and decision making in a specialized field (major groups 1 and 2) were coded as skill level 3–4 (high/specialized). Finally, and in order to facilitate the analysis and interpretation of the results, these skill levels were dichotomized according to the degree of cognitive demands or skill levels with 1 = low-middle skill level occupations (including skill levels 1 and 2) and 2 = middle-high skill level occupations (skill levels 3 and 4).

Furthermore, given that occupation is related to levels of education, and that both socio-economic indicators may have synergistic effects on late-life health (Then et al., 2014; Yuan et al., 2015), joint effects of the skill level occupations and the level of education were analyzed. To this end, the following combinations were created: “low and low” (occupations with low-middle requirements and less than 8 years of education), “low and middle OR high and low” (low-middle requirements and 9–17 years of education, or middle-high requirements and less than 8 years of education), “low and high OR high and middle” (low-middle requirements and more than 17 years of education, or middle-high requirements and 9–17 years of education), and “high and high” (middle-high requirements and more than 17 years of education).

### Statistics

All statistical analyses were performed using the PASW Statistics 18 statistical package version 18.0.0 (PASW, 2009). The level of significance was defined as *P* < 0.05. The main independent variables were the DEGURBA and the skill level of principal occupation. The normality assumption, tested with Kolmogorov–Smirnov test, was not justified, but the sample size was sufficiently large to apply parametric instead of nonparametric tests. Comparisons between variables were made using Pearson’s chi-square tests for dichotomous variables and Student’s *t*-tests for continuous variables. Separate multiple logistic regression analyses were conducted to develop a model for testing if the DEGURBA and main lifetime occupation significantly predicted cognitive impairment. In a second step, age, sex, and level of education were introduced in the models as predictors, as these have been previously identified as risk factors for cognitive impairment and dementia.

## Results

### Prevalence of Cognitive Impairment According to the Degree of Urbanization

The socio-demographic characteristics, MMSE average scores, and distribution of the prevalence of cognitive impairment as a function of the DEGURBA are summarized in **Table 2**.

**Table 2:** Socio-demographic characteristics, MMSE average scores, and prevalence rates for cognitive impairment as a function of the degree of urbanization (DEGURBA).

	DPA (*n* = 375)	ITPA (*n* = 374)	*P*-value
Age, mean (*SD*)	76.33 (7.11)	75.19 (7.17)	0.029^∗^
Sex, *n* (%)			0.080
Female	239 (63.7)	215 (57.5)	
Male	136 (36.3)	159 (42.5)	
Education, *n* (%)			<0.0001^∗^
≤8 years	168 (44.8)	283 (75.7)	
9–17 years	118 (31.5)	61 (16.3)	
>17 years	89 (23.7)	30 (8.0)	
MMSE, mean (*SD*)	28.38 (2.24)	28.21 (2.38)	0.314
Cognitive impairment, *n* (%)	20 (5.3)	29 (7.8)	0.180


*DPA, densely populated areas; ITPA: intermediate-thinly populated areas; MMSE, Mini-Mental State Examination; SD, standard deviation. ^∗^*P* < 0.05 (*t*-test for means and χ^2^ for percentages).*

In the DPA (*n* = 375) the average age was 76.33 years (*SD* = 7.11) and 63.7% were female, while in the ITPA (*n* = 374) the average age was 75.19 years (*SD* = 7.17) and 57.5% were female (see **Table 1**). Groups were homogeneous in sex [χ^2^(1, *n* = 749) = 3.06, *P* = 0.080]. The DPA participants were significantly older than the ITPA participants [*t*(747) = 2.190, *P* = 0.029], and in the rural areas the participants had a lower education level [χ^2^(2, *n* = 749) = 76.72, *P* < 0.0001]. Thus, 75.7% of ITPA participants studied ≤8 years of formal education and only 8.0% studied >17 years, compared to DPA with 44.8% of people with ≤8 years of education, and 23.7% of people with >17 years.

**FIGURE 2:**
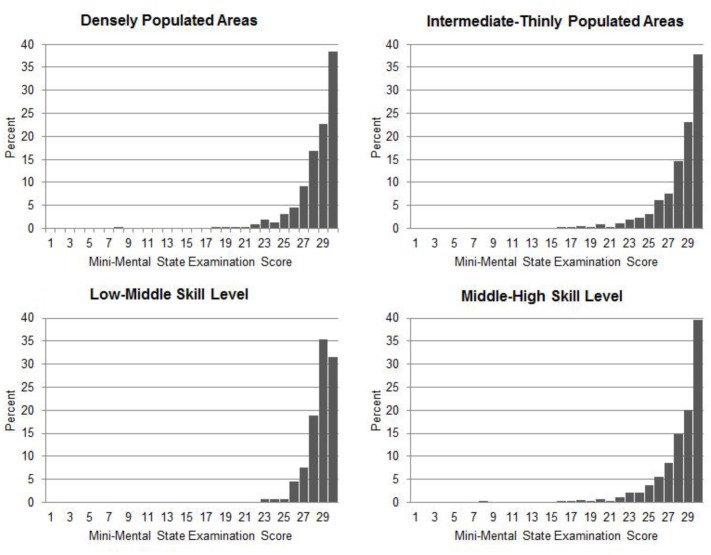
**Distribution of Mini-Mental State Examination (MMSE) scores as a function of degree of urbanization (DEGURBA) (upper-row) and skill level (lower-row)**.

The MMSE average scores adjusted by age and level of education are shown in **Table 2** according to the urbanization degree. The average score in the DPA was 28.38 (*SD* = 2.24) and ranged from 8 to 30, and in the ITPA was 28.21 (*SD* = 2.38) and ranged from 16 to 30 (see **Figure 2**, upper row). As shown in the figure, both groups showed clustering at high scores. Student’s *t*-test revealed no significant differences between the MMSE average scores in urban and rural populations [*t*(747) = 1.008, *P* = 0.314].

Based on the screening criteria, the overall prevalence of cognitive impairment in the studied sample was 6.5% [7.9% in females, 4.4% in males, χ^2^(1, *n* = 749) = 3.63, *P* = 0.057]. The prevalence of cognitive impairment in the DPA group was 5.3%, and for ITPA group was 7.8%, although chi-square tests revealed that the prevalence of cognitive impairment was not significantly different between urban and rural populations [χ^2^(1, *n* = 749) = 1.79, *P* = 0.180].

### Relation between Principal Lifetime Occupation and General Cognitive Performance

Data on occupation was missing for three participants who described their occupational title as “emigrant.” 84.2% were retired individuals. Most of the participants (82.2%, *n* = 613) had occupations requiring low-middle skill levels, and 17.8% (*n* = 133) had occupations with middle-high skill levels.

**Table 3** shows the socio-demographic characteristics, MMSE average scores, and distribution of the prevalence of cognitive impairment according to the occupational groups. As it can be seen in **Table 3**, groups were homogeneous in sex [χ^2^(1, *n* = 746) = 0.096, *P* = 0.756], but there were significant differences in age [participants in occupations with low-middle requirements were older; *t*(744) = 1.979, *P* = 0.048], and in years of education [χ^2^(2, *n* = 746) = 211.85, *P* < 0.0001]. Specifically, within the group of participants in occupations with low-middle requirements, 69.0% had ≤8 years of formal education, whereas in the group with occupations with middle-high skill levels, 56.4% had >17 years of education.

**Table 3:** Socio-demographic characteristics, MMSE average scores, and prevalence rates for cognitive impairment as a function of the occupations’ skill level.

	Low-middle (*n* = 613)	Middle-high (*n* = 133)	*P*-value
Age, mean (*SD*)	76.02 (7.13)	74.67 (7.19)	0.048^∗^
Sex, *n* (%)			0.756
Female	373 (60.8)	79 (59.4)	
Male	240 (39.2)	54 (40.6)	
Education, *n* (%)			<0.0001^∗^
≤8 years	423 (69.0)	25 (18.8)	
9–17 years	146 (23.8)	33 (24.8)	
>17 years	44 (7.2)	75 (56.4)	
MMSE, mean (*SD*)	28.20 (2.47)	28.74 (1.31)	0.016^∗^
Cognitive impairment, *n* (%)	47 (7.7)	2 (1.5)	0.009^∗^


*MMSE, Mini-Mental State Examination; SD, standard deviation. ^∗^*P* < 0.05 (*t*-test for means and χ^2^ for percentages).*

As shown in **Table 3**, the MMSE average score in the group with low-middle requirements was 28.20 (*SD* = 2.47) and ranged from 23 to 30, and in the group with occupations with middle-high skill levels was 28.74 (*SD* = 1.32) and ranged from 8 to 30 (see **Figure 2**, lower row), with both groups showing clustering at high scores. Student’s *t*-test revealed that cognitive performance in the MMSE significantly differed between the occupational groups [*t*(744) = -2.415, *P* = 0.016], with higher scores in the group with middle-high cognitive requirements.

Results of logistic regression analyses revealed a statistically significant association between skill level of principal lifetime occupation and prevalence of cognitive impairment (OR = 0.18; 95% CI = 0.04–0.77, β = -1.69, *P* = 0.020). Specifically, within the group of participants in occupations with low-middle requirements the prevalence of cognitive impairment was 7.7%, whereas in the group with middle-high requirements the prevalence of cognitive impairment was significantly lower, 1.5% [χ^2^(1, *n* = 746) = 6.76, *P* = 0.009]. In order to explore this result in more detail we have estimated the prevalence of cognitive impairment for each skill level separately (1–4). Prevalences reduced progressively from basic to specialized skill levels [skill level 1: 16.2%, skill level 2: 7.1%, skill level 3: 2.3%, skill level 4: 1.1%, χ^2^(3, *n* = 746) = 11.52, *P* = 0.009]. Having had occupations with low-middle requirements was a risk factor for cognitive impairment. However, this association did not remain significant after adjustment for age, sex, and level of education. Basic descriptive statistics and regression coefficients are shown in **Table 4**. The Wald criterion demonstrated that only age (β = -0.04, *P* = 0.043) and level of education (β = 1.43, *P* = 0.001) had significant partial effects in the full model, being significant predictors of cognitive impairment. Specifically, the prevalence of cognitive impairment increased with age and in persons with lower levels of formal education.

**Table 4:** Association between cognitive impairment and skill level of principal lifetime occupation after adjustment for age, sex, and level of education.

	OR (95% CI)	*P*-value
Skill level	0.59 (0.13–2.63)	0.487
Age	0.96 (0.92–0.99)	0.043^∗^
Sex	1.64 (0.84–3.20)	0.148
Education	4.20 (1.7–10.06)	0.001^∗^


*OR, odds ratio; CI, confidence interval to ^∗^*P* < 0.05.*

As stated, given that occupation is related to levels of education, and that both indicators may have synergistic effects, we finally analyzed joint effects of the occupations skill level and the level of education. The cognitive impairment prevalence rates by the “skill level of occupation and education” are shown in **Table 5**. The number of persons in the low-low category was higher (*n* = 423, 56.7%) in comparison with the other categories. A significant additive effect of skill level and education was observed on cognitive impairment [χ^2^(3, *n* = 746) = 23.88, *P* < 0.0001] (see **Table 5**).

**Table 5:** Prevalence of cognitive impairment by combinations of occupations skill level and education.

	Total, *n* (%)	Cognitive impairment	*P*-value
Skill Level and Education			<0.0001^∗^
Low and Low	423 (56.7)	44 (10.4)	
Low and Middle OR High and Low	171 (22.9)	4 (2.3)	
Low and High OR High and Middle	77 (10.3)	0 (0.0)	
High and High	75 (10.1)	1 (1.3)	


*Low and Low: occupations with low-middle requirements and ≤8 years of education; Low and Middle OR High and Low: low-middle requirements and 9–17 years of education or middle-high requirements and ≤8 years of education; Low and High OR High and Middle: low-middle requirements and ≥17 years of education or middle-high requirements and 9–17 years of education; High and High: middle-high requirements and ≥17 years of education.^∗^*P* < 0.05.*

An interesting finding was that the prevalence of cognitive impairment was higher in participants having had occupations with low-middle requirements and with fewer years of formal education (“low and low” category) compared with participants in “high and high” category. In categories that deviated from the expected pattern (“low and middle OR high and low”, “low and high OR high and middle”) the prevalence of cognitive impairment was very low or inexistent.

## Discussion

This is the largest study providing estimates of the real prevalence rates and covariates of cognitive impairment in Galician elderly populations in two defined geographic areas, densely populated areas, and intermediate-thinly populated areas. This approach allowed us to directly compare the prevalence of cognitive impairment in urban and rural populations and their specific risk factors. Another purpose of the present study was to examine the association between the skill level of principal lifetime occupation and current general cognitive performance as measured by the MMSE.

### Prevalence of Cognitive Impairment According to the Degree of Urbanization

In the present study, it was found that 6.5% of older adults aged ≥65 participating in senior centers were cognitively impaired; 5.3% in DPA and 7.8% in ITPA. These prevalence estimates were relatively lower than previously reported (22.2%) in a cross-sectional study including a representative sample of 600 community-dwellers over 65 residents of Narón Council (Galicia, Spain) (Millán-Calenti et al., 2009). The estimation of prevalence was also lower than in other Spanish councils (Graciani et al., 2006), and in other European countries (approximately 18%, Brayne et al., 1998; Rait et al., 2005). The variability seen in these studies may in large part be attributed to differences in participant samples. VERISAÚDE population included older adults attending senior centers. In this regard, community and senior centers have become one of the most widely used services among older adults both in urban and rural regions and they generally offer a wide variety of programs and services, including social and recreational activities. It is possible that older adults who regularly and actively participate in these activities may have higher levels of health, social interaction, and quality of life (Bugallo Carrera et al., 2014), which can help them stay healthy and maintain cognitive function.

Degree of urbanization was not significantly associated with risk of cognitive impairment in the present study. In fact, the prevalence of cognitive impairment did not significantly differ between the geographical areas explored. However, in accordance with previous studies (Nunes et al., 2010), a trend was observed to a higher prevalence of cognitive impairment in rural compared to urban populations, despite urban residents were significantly older than rural residents. This may be explained by the educational level that was lower in rural areas. It is important to note, however, that our findings are based on a measure of global cognitive status (MMSE), and we cannot rule out the possibility that DEGURBA is related to cognitive impairment in some cognitive domains but not in others. Importantly, although frequently used in clinical research, a potential lack of sensitivity for cognitive decline of this screening instrument has been previously reported. In fact, it has a ceiling effect (as observed in the present study), which limits the detection of dementia in well-educated populations (Reisberg et al., 1997). A potential limitation of this study is that only present residence place was considered; people participating in senior centers at urban and rural areas were evaluated, but early adulthood or childhood residence was not taken into account. In this line, it has been shown that urban residence at early life may be protective against having cognitive impairment in the future, independently of education (Zhang et al., 2008).

### Relation between Principal Lifetime Occupation and General Cognitive Performance

One of our main objectives was to determine whether the lifetime longest-held occupation is involved in the processes leading to cognitive impairment. In this regard, a significant relationship between the skill level of occupations and cognitive impairment was found in the present study. In fact, the prevalence of cognitive impairment was significantly lower within the group of participants having had occupations with higher requirements. Consistent with earlier studies (Jorm et al., 1998), the lowest MMSE scores and the highest prevalence of cognitive impairment were found in the occupations involving the lower use of cognitive skills. In this line, it has been proposed that higher levels of education and occupations with high cognitive requirements possibly allow older people to use cognitive processing or compensatory strategies to cope better with age-related cognitive decline (they experience a higher level of cognitive reserve; Stern, 2002). A recent magnetoencephalography (MEG) study revealed that subjects with low cognitive reserve need a greater effort than those with high cognitive reserve to successfully perform the same cognitive task, in terms of functional connectivity (López et al., 2014). Our finding is in line with a previous longitudinal study showing that low levels of education and low lifetime occupational attainment are associated with increased risk of cognitive impairment and dementia in a Spanish elderly cohort (Alvarado et al., 2002).

However, this protective effect was not independent of confounding factors, such as age and education level, in the present study. Higher prevalence of cognitive impairment was associated with being older and having lower levels of education. In fact, the association between skill level and cognitive impairment was greatly mediated by education level. A significant additive effect of the skill level of occupation and education level was observed on cognitive impairment, being the prevalence of cognitive impairment higher in participants having had occupations with low-middle cognitive requirements and with lower levels of formal education. These results agree with previous studies suggesting that having had an occupation with high mental demands and having a high educational level is associated with a better cognitive functioning in elderly (Frisoni et al., 1993; Jones and Gallo, 2002; Bosma et al., 2003; Staff et al., 2004; Andel et al., 2005; Then et al., 2014), providing a form of mental protection that supports brain function, facilitating the maintenance of cognitive reserve and promoting stable cognitive function. Higher complexity of main lifetime occupation and cognitively demanding work conditions have been also associated with a decreased risk of dementia and cognitive decline in old age even after controlling for age, sex, and level of education (Andel et al., 2005, 2011; Then et al., 2014).

Importantly, in a recent longitudinal study (Zahodne et al., 2011), it was observed that education was related to cognitive performance but it did not slow cognitive decline with aging. Thus, longitudinal studies are necessary to establish robust conclusions on this topic.

To sum up, older adults with fewer years of formal education and having had less skilled occupations showed greater declines in cognitive function, independently of the DEGURBA of the present residence place.

The main strength of the present research is that this is the largest multicenter study providing estimates of the real prevalence rates of cognitive impairment in Galician elderly populations in rural and urban areas, and examining the association between the skill level of principal lifetime occupation and education and current general cognitive performance. The main limitations, however, are the cross sectional nature of the design, the sample selection in senior centers, and the fact that the sample was not representative in terms of educational levels, which could affect the generalization of the findings. Future longitudinal studies are needed to further assess causal relationships and to disentangle the synergistic influence of occupation and education on age-related cognitive impairment.

## Conclusion

Area of present residence did not significantly affect cognitive impairment in the present study. Our findings suggest, however, a significant association between lifetime longest-held occupations skill level and better cognitive functioning in later life. In fact, older adults who had held occupations with higher skill levels had a lower risk of cognitive impairment. However, this protective effect was greatly dependent on age and educational level. This result suggests that occupation during active life is not an isolated protective factor against cognitive impairment, and it is closely related to educational level. In future preventive geriatric interventions both in rural and urban areas, description of both occupation and educational level should be taken into consideration in screening older adults at increased risk of cognitive impairment and dementia, and special priority must be given to low-educated older adults, as they are more vulnerable to impaired cognitive function. It is important to highlight that only active older participants in senior centers were assessed in the present study, possibly affecting the generalization of the findings. However, this kind of results may have important implications to social policy since increasing reserve in adulthood and midlife could have an important clinical value in the future, possibly delaying the clinical manifestations of age-related diseases, such as dementia in future older generations.

## Ethics Statement

The study protocol was approved by the Ethics Committee of the University of A Coruña and was in conformity with the principles embodied in the Declaration of Helsinki. Before the data collection, all participants were informed about the study and signed the corresponding informed consent form.

## Author Contributions

All authors meet all four criteria for authorship recommended by the International Committee of Medical Journal Editors and agree to be accountable for the content of the work. AM, JM-C, and LL-L contributed to the conception and design of the work, analysis and interpretation of data. LL-L, RL-L, and CD-D contributed to the data acquisition. JM-C, AM, LL-L, BL, EP, and VV collaborated in the interpretation of data. LL-L drafted the work. All authors participated in revising it critically for important intellectual content and gave final approval of the version to be published.

## Conflict of Interest Statement

The authors declare that the research was conducted in the absence of any commercial or financial relationships that could be construed as a potential conflict of interest.

## References

[B1] Alvarado B. E. Zunzunegui M. V. Del Ser T. Béland F. (2002). Cognitive decline is related to education and occupation in a Spanish elderly cohort. *Aging Clin. Exp. Res.* 14 132–142. 10.1007/bf03324426 12092786

[B2] Andel R. Crowe M. Kåreholt I. Wastesson J. Parker M. G. (2011). Indicators of job strain at midlife and cognitive functioning in advanced old age. *J. Gerontol. B Psychol. Sci. Soc. Sci.* 66 287–291. 10.1093/geronb/gbq105 21292810

[B3] Andel R. Crowe M. Pedersen N. L. Mortimer J. Crimmins E. Johansson B. (2005). Complexity of work and risk of Alzheimer’s Disease: a population-based study of Swedish twins. *J. Gerontol. B Psychol. Sci. Soc. Sci.* 60B, 251–258. 10.1093/geronb/60.5.P251 16131619

[B4] Baldivia B. Andrade V. M. Amodeo Bueno O. F. (2008). Contribution of education, occupation and cognitively stimulating activities to the formation of cognitive reserve. *Dement. Neuropsychol.* 2 173–182. 10.1590/S1980-57642009DN20300003 PMC5619462 29213567

[B5] Blesa R. Pujol M. Aguilar M. Santacruz P. Bertran-Serra I. Hernández G. (2001). Clinical validity of the “mini-mental state” for Spanish speaking communities. *Neuropsychologia* 39 1150–1157. 10.1016/s0028-3932(01)00055-0 11527552

[B6] Bosma H. van Boxtel M. P. Ponds R. W. Houx P. J. Burdorf A. Jolles J. (2003). Mental work demands protect against cognitive impairment: MAAS prospective cohort study. *Exp. Aging Res.* 29 33–45. 10.1080/03610730303710 12735080

[B7] Brayne C. Nickson C. McCracken C. (1998). Cognitive function and dementia in six areas of England and Wales: the distribution of MMSE and prevalence of GMS organicity level in the MRC CFA Study. The Medical Research Council Cognitive Function and Ageing Study (MRC CFAS). *Psychol. Med.* 28 319–335. 10.1017/s0033291797006272 9572090

[B8] Bugallo Carrera C. Gandoy Crego M. Gómez Cantorna C. (2014). La calidad de vida de los sujetos usuarios de un centro social de personas mayores. [The quality of life of the users of a community center for elderly]. *Gerokomos* 25 103–106. 10.4321/s1134-928x2014000300004

[B9] Correa Ribeiro P. C. Lopes C. S. Lourenço R. A. (2013). Complexity of lifetime occupation and cognitive performance in old age. *Occup. Med.* 63 556–562. 10.1093/occmed/kqt115 24253807

[B10] Eurostat Task Force on Core Social Variables (2007). *Final Report.* Available at: http://ec.europa.eu/eurostat/documents/3859598/5901513/KS-RA-07-006-EN.PDF/71481ffb-771a-489b-a749-1a055c0247d4 [accessed January 13].

[B11] Fisher G. G. Stachowski A. Infurna F. J. Faul J. D. Grosch J. Tetrick L. E. (2014). Mental work demands, retirement, and longitudinal trajectories of cognitive functioning. *J. Occup. Health Psychol.* 19 231–242. 10.1037/a0035724 24635733 PMC4663987

[B12] Folstein M. F. Folstein S. E. McHugh P. R. (1975). “Mini-Mental State”. A practical method for grading the cognitive state of patients for the clinician. *J. Psychiatr. Res.* 12 189–198. 10.1016/0022-3956(75)90026-6 1202204

[B13] Frisoni G. B. Rozzini R. Bianchetti A. Trabucchi M. (1993). Principal lifetime occupation and MMSE score in elderly persons. *J. Gerontol. Soc. Sci.* 48 S310–S314. 10.1093/geronj/48.6.S310 8228005

[B14] Fritsch T. McClendon M. J. Smyth K. A. Lerner A. J. Friedland R. P. Larsen J. D. (2007). Cognitive functioning in healthy aging: the role of reserve and lifestyle factors early in life. *Gerontologist* 47 307–322. 10.1093/geront/47.3.307 17565095

[B15] Galician Statistics Institute (2011). *Classification of Municipalities According to the Degree of Urbanization*. Available at: http://www.ige.eu/estatico/pdfs/s3/clasificacions/urbanizacion/NotasMetodoloxicas_Rev.pdf [accessed January 13].

[B16] Graciani A. Banegas J. R. Guallar-Castillon P. Domínguez-Rojas V. Rodríguez-Artalejo F. (2006). Cognitive assessment of non-demented elderly community dwellers in Spain. *Dement. Geriatr. Cogn. Disord.* 21 104–112. 10.1159/000090509 16374005

[B17] International Labour Organization [ILO] (2012). *International Standard Classification of Occupations 2008 (ISCO-08)*. http://www.ilo.org/wcmsp5/groups/public/—dgreports/—dcomm/—publ/documents/publication/wcms_172572.pdf [accessed January 13].

[B18] Jones R. N. Gallo J. J. (2002). Education and sex differences in the Mini-Mental State Examination: effects of differential item functioning. *J. Gerontol. B Psychol. Sci. Soc. Sci.* 57 548–558. 10.1093/geronb/57.6.p548 12426438

[B19] Jorm A. F. Henderson A. Scott R. Korten A. Christensen H. Mackinnon A. (1994). Does education protect against cognitive impairment? A comparison of the elderly in two Australian cities. *Int. J. Geriatr. Psychiatry* 9 357–363. 10.1002/gps.930090503

[B20] Jorm A. F. Rodgers B. Henderson A. S. Korten A. E. Jacomb P. A. Christensen H. (1998). Occupation type as a predictor of cognitive decline and dementia in old age. *Age Ageing* 27 477–483. 10.1093/ageing/27.4.477 9884005

[B21] Keefover R. W. Rankin E. D. Keyl P. M. Wells J. C. Martin J. Shaw J. (1996). Dementing illnesses in rural populations: the need for research and challenges confronting investigators. *J. Rural Health* 12 178–187. 10.1111/j.1748-0361.1996.tb00792.x 10172872

[B22] Lojo-Seoane C. Facal D. Guàrdia-Olmos J. Juncos-Rabadán O. (2014). Structural model for estimating the influence of cognitive reserve on cognitive performance in adults with subjective memory complaints. *Arch. Clin. Neuropsychol.* 29 245–255. 10.1093/arclin/acu007 24663239

[B23] López M. E. Aurtenetxe S. Pereda E. Cuesta P. Castellanos N. P. Bruña R. (2014). Cognitive reserve is associated with the functional organization of the brain in healthy aging: a MEG study. *Front. Aging Neurosci.* 6:125 10.3389/fnagi.2014.00125 PMC4056015 24982632

[B24] Martín Lesende I. García Rodríguez A. Abajo Angulo J. L. Olabarría Ateca V. González García J. Rueda Alonso E. (2001). Comparison of the health care status with a global geriatric assessment in a ≥ 75 year old rural and urban population within the same health care area. *Rev. Esp. Geriatr. Gerontol.* 36 150–155. 10.1016/S0211-139X(01)74706-3

[B25] Millán-Calenti J. C. Tubío J. Pita-Fernández S. González-Abraldes I. Lorenzo T. Maseda A. (2009). Prevalence of cognitive impairment: effects of level of education, age, sex and associated factors. *Dement. Geriatr. Cogn. Disord.* 28 455–460. 10.1159/000257086 19907183

[B26] Millán-Calenti J. C. Tubío J. Pita-Fernández S. Rochette S. Lorenzo T. Maseda A. (2012). Cognitive impairment as predictor of functional dependence in an elderly sample. *Arch. Gerontol. Geriatr.* 54 197–201. 10.1016/j.archger.2011.02.010 21397345

[B27] National Statistics Institute (2011). *Population Demographic Censuses. Municipal Register* 2011. Madrid: INE.

[B28] National Statistics Institute (2012). *National Classification of Occupations (CNO2011)*. Available at: http://www.ine.es/daco/daco42/clasificaciones/Introduccion_CNO11.V02.pdf [accessed January 13].

[B29] Nunes B. Silva R. D. Cruz V. T. Roriz J. M. Pais J. Silva M. C. (2010). Prevalence and pattern of cognitive impairment in rural and urban populations from Northern Portugal. *BMC Neurol.* 10:42 10.1186/1471-2377-10-42 PMC2905352 20540726

[B30] PASW (2009). *PASW^®^ Statistics 18 Core System User’s Guide.* Available at: http://www.unt.edu/rss/class/Jon/SPSS_SC/Manuals/v18/PASW%20Statistics%2018%20Core%20System%20User’s%20Guide.pdf [accessed January 14].

[B31] Rait G. Fletcher A. Smeeth L. Brayne C. Stirling S. Nunes M. (2005). Prevalence of cognitive impairment: results from the MRC trial of assessment and management of older people in the community. *Age Ageing* 34 242–248. 10.1093/ageing/afi039 15863409

[B32] Reisberg B. Burns A. Brodaty H. Eastwood R. Rossor M. Sartorius N. (1997). Diagnosis of Alzheimer’s disease: report of an international psychogeriatric association meeting work group. *Int. Psychogeriatr.* 9 11–38. 10.1017/S1041610297004663 9447425

[B33] Sando S. B. Melquist S. Cannon A. Hutton M. Sletvold O. Saltvedt I. (2008). Risk-reducing effect of education in Alzheimer’s disease. *Int. J. Geriatr. Psychiatry* 23 1156–1162. 10.1002/gps.2043 18484674

[B34] Scarmeas N. Stern Y. (2003). Cognitive reserve and lifestyle. *J. Clin. Exp. Neuropsychol.* 25 625–633. 10.1076/jcen.25.5.625.14576 12815500 PMC3024591

[B35] Staff R. T. Murray A. D. Deary I. J. Whalley L. J. (2004). What provides cerebral reserve? *Brain* 127 1191–1199. 10.1093/brain/awh144 15047587

[B36] Steffens D. C. McQuoid D. R. Potter G. G. (2014). Amnestic mild cognitive impairment and incident dementia and Alzheimer’s disease in geriatric depression. *Int. Psychogeriatr.* 26 2029–2036. 10.1017/S1041610214001446 25032667 PMC4227924

[B37] Stern Y. (2002). What is cognitive reserve? Theory and research application of the reserve concept. *J. Int. Neuropsychol. Soc.* 8 448–460. 10.1017/s1355617702813248 11939702

[B38] Stern Y. (2012). Cognitive reserve in ageing and Alzheimer’s disease. *Lancet Neurol.* 11 1006–1012. 10.1016/S1474-4422(12)70191-6 23079557 PMC3507991

[B39] Then F. S. Luck T. Luppa M. Arélin K. Schroeter M. L. Engel C. (2014). Association between mental demands at work and cognitive functioning in the general population–Results of the health study of the Leipzig research center for civilization diseases (LIFE). *J. Occup. Med. Toxicol.* 9 23 10.1186/1745-6673-9-23 PMC4049483 24914403

[B40] United Nations Department of Economic Social Affairs Population Division (2015). *World Population Prospects: The 2015 Revision, Key Findings and Advance Tables. Working Paper No. ESA/P/WP.* New York, NY: United Nations, 241.

[B41] Vandenbroucke J. P. von Elm E. Altman D. G. Gøtzsche P. C. Mulrow C. D. Pocock S. J. (2014). Strengthening the reporting of observational studies in epidemiology (STROBE): explanation and elaboration. *Int. J. Surg.* 12 1500–1524. 10.1016/j.ijsu.2014.07.014 25046751

[B42] von Elm E. Altman D. G. Egger M. Pocock S. J. Gøtzsche P. C. Vandenbroucke J. P. (2014). The Strengthening the reporting of observational studies in epidemiology (STROBE) statement: guidelines for reporting observational studies. *Int. J. Surg.* 12 1495–1499. 10.1016/j.ijsu.2014.07.013 25046131

[B43] Yuan M. Chen W. Chu C. I. Fang Y. (2015). Joint effect of education and main lifetime occupation on late life health: a cross-sectional study of older adults in Xiamen, China. *PLoS ONE* 10 e0131331 10.1371/journal.pone.0131331 PMC4480357 26107636

[B44] Zahodne L. B. Glymour M. M. Sparks C. Bontempo D. Dixon R. A. MacDonald S. W. S. (2011). Education does not slow cognitive decline with aging: 12-year evidence from the Victoria Longitudinal Study. *J. Int. Neuropsychol. Soc.* 17 1039–1046. 10.1017/S1355617711001044 21923980 PMC3285821

[B45] Zhang Z. Gu D. Hayward M. D. (2008). Early life influences on cognitive impairment among oldest old Chinese. *J. Gerontol. B Psychol. Sci. Soc. Sci.* 63B, S25–S33. 10.1093/geronb/63.1.s25 18332198

